# Pain assessment tools for use in infants: a meta-review

**DOI:** 10.1186/s12887-023-04099-7

**Published:** 2023-06-19

**Authors:** Diana Arabiat, Evalotte Mörelius, Kreshnik Hoti, Jeffery Hughes

**Affiliations:** 1 Maternal and Child Nursing Department, Faculty of Nursing, The University of Jordan, Amman, Jordan; 2 School of Nursing and Midwifery, Edith Cowan University, Joondalup, Western Australia Australia; 3 Department of Health, Medicine and Caring Sciences, Linköping University, Linköping, Sweden; 4 Faculty of Medicine, University of Prishtina, Pristina, Kosovo; 5 Curtin Medical School, Faculty of Health Sciences, Curtin University, Bentley, WA Australia

**Keywords:** Infants, Validity, Reliability, Clinical utility, Observational pain assessment tools

## Abstract

**Background:**

Identifying pain in infants is challenging due to their inability to self-report pain, therefore the availability of valid and reliable means of assessing pain is critical.

**Objective:**

This meta-review sought to identify evidence that could guide the selection of appropriate tools in this vulnerable population.

**Methods:**

We searched Scopus, Medline, Embase, CINAHL, MIDRIS, EMCare and Google Scholar for eligible systematic reviews. Eligible reviews documented psychometric properties of available observational tools used to assess pain in infants.

**Results:**

A total of 516 reviews were identified of which 11 met our inclusion criteria. We identified 36 pain assessment tools (evaluated in 11 reviews) of which seven were reported in at least three reviews. The level of evidence reported on the psychometric properties of pain assessment tools varied widely ranging from low to good reliability and validity, whilst there are limited data on usability and clinical utility.

**Conclusions:**

Currently, no observer administered pain assessment tool can be recommended as the gold standard due to limited availability and quality of the evidence that supports their validity, reliability and clinical utility. This meta-review attempts to collate the available evidence to assist clinicians to decide on what is the most appropriate tool to use in their clinical practice setting. It is important that researchers adopt a standard approach to evaluating the psychometric properties of pain assessment tools and evaluations of the clinical utility in order that the highest level of evidence can be used to guide tool selection.

**Supplementary Information:**

The online version contains supplementary material available at 10.1186/s12887-023-04099-7.

## Background

Pain occurs across the continuum of life. However, for those who cannot self-report their pain, such as pre-verbal children, those with intellectual disability and those with neurological disorders who have lost the ability to communicate (e.g., people living with advanced dementia), there is evidence that their pain often goes under-recognized and under-treated [1–3]. Research clearly indicates that accurate pain assessment in infants is a challenge for health care professionals [2, 4]. The problem of differentiating or discriminating pain from other expressions of unpleasant feelings or experiences, such as hunger, fear or distress adds to the complexity of assessing pain in this group [5].

In the absence of infants’ ability to self-report, observation of behaviours such as facial expression, crying, hand and leg movements is a valid approach for pain assessment [3, 4, 6]. However, pain behaviours are not specific reflections of pain intensity or distress, and the context of infant’s behaviour must be considered when using this approach [6].

Herr et al. [6], report that typically children as young as 3 years may be able to report pain using basic tools, however it is not until 8 years of age that children are generally considered reliable in using a numerical rating scale. In clinical practice, Eccleston et al. [7], in support of Beltramini et al. [8], suggest that self-reported measures of pain (e.g., numerical scales) can be used for children aged 6 years and older, while behavioral scales can be used for children aged below 6 years or those unable to verbally report. According to the American Society of Pain Management Nursing (APSMN) position statement on pain assessment in the patient unable to self-report, health care professionals should search for potential causes of pain and use both primary behavioral categories and behavioral pain tools for initial and ongoing assessment of pain [6].

An essential element in assessment and management of pain is the identification of the type and source of pain, both of which can be challenging in the infant population [8]. The following types of pain have been identified in infants: acute pain that occurs in response to recent illness, injury or following procedures or operations, and has a limited duration; and chronic pain which commonly persists beyond the time of curing of an injury or illness, and often lasts beyond 6 months [9]. Validated assessment tools in infants have been developed mostly within research into acute pain. These included comprehensive identification of primary behavioral categories, such as facial expression, body activity/motor movement, and crying/verbalization [2]. For assessment of chronic pain, infants may demonstrate different behaviours, such as withdrawal, lack of expression, lack of interest in surroundings, and a decrease in their ability or their willingness to play [10].

Eccleston et al, [7] proposed four transformative goals to improve the lives of children and adolescents with pain and their families. These were to 1) “make pain matter”, 2) “make pain understood”, 3) “make pain visible”, and 4) “make pain better”. They pointed out that whilst on face value these goals might seem obvious and simple, the ongoing occurrence of acute pain, pain post-surgery and procedures, and chronic pain in this population suggest otherwise. Considering the above goals highlighted by Eccleston et al,[7] of particular importance remains the issue of adequately identifying and therefore assessing pain using evidence-based tools. In this regard, previous reviews have identified that while there are a number of tools that can be used in infant population, the strength of psychometric evidence to support their use varies [8, 11],. Therefore, there is an ongoing need to review evidence and therefore progress made in this area. Meta-reviews, also known as systematic reviews of systematic reviews, provide a rigorous approach for synthesizing evidence from available systematic reviews and summarizing knowledge into one accessible document [12]. Given the wide variety of pain assessment tools and uncertainty as to which assessment tools are more suitable when used in clinical or multiple settings and within different populations, we felt that a meta-review was particularly useful for our purpose. Undertaking this meta-review would provide an updated systematic identification and appraisal of the evidence concerning the validity and reliability of pain assessment tools available for use in infants aged 1-12 months old. This would then allow an evaluation of the appropriateness of these pain tools for use in different clinical settings. This age range was selected in order to exclude tools which are used exclusively for pre-term and full-term neonates.

## Methods

### Design

We performed a systematic literature review of published reviews in accordance with a protocol that was published in advance on the PROSPERO database with reference number CRD42021236227. We used the Joanna Briggs Institute (JBI) methodology for systematic review of systematic reviews [12] and the Preferred Reporting Items for Systematic Review [13] and Meta-Analysis (PRISMA-s) guideline for conducting and reporting our findings [14].

### *Reviewquestionandeligibility*

This meta-review aimed to address the following questions: 1) What pain assessment tools are available for assessing pain in infants; 2) What are the documented psychometric properties of the available tools in this population; and 3) How have previous reviews evaluated and recommended these pain tools for use in infants?

Studies needed to meet the following inclusion criteria to be eligible for inclusion in this review (the formulation of the research question with reference to PICO (Patient/Population, Intervention, Comparison and Outcomes) is provided in the Supplementary Table 1):

1. Participants: This meta-review considered reviews that report on the pain assessment tools used for infants, of either gender. Infant in this review is defined as any child aged between 1 and 12 months of age. Children less than 1 month were considered neonates [15], and tools used exclusively to assess this population were excluded.
2. Exposure of interest: The exposure of interest is pain assessment tools. Reviews that include management of pain were considered if they have also covered assessment of pain. The International Association for the Study of Pain (IASP) suggested that pain assessments entail a comprehensive evaluation of the patient’s pain, symptoms, functional status, and clinical history [16]. According to Stevens et al. [17], acute pain is often limited to a short period of time and in infants is frequently associated with procedures (e.g. venipuncture, immunization, wound dressings), trauma (e.g. burns), or post-surgery, while chronic pain persists beyond 3 – 6 months and may either be recurrent as is the case with headache, backache, stomach aches or prolonged as that associated with use of ventilators for an extended period of time. For this review, all forms of pain were considered.
3. Outcomes: This meta-review considered reviews that included pain assessment tools irrespective of the outcomes of the assessment (e.g., infant being in pain or not).
4. Types of studies: This meta-review considered all type of reviews including integrative, scoping, and meta-analysis studies that have evaluated the validity and reliability of pain assessment tools used in infants.

Reviews of studies of the assessment of pain and pain assessment tools were regarded as valid for the purpose of this meta-review if the conditions listed below were met: (1) the review carried out systematically (e.g., publication that makes explicit the authors’ intention to review or summarize the literature; with review, overview, or meta-analysis in the title or in the section heading); (2) entails a clear set of objectives (explicit and clear research question); (3) reproducible methodology (the paper clearly explains how the evidence was retrieved, including sources and search strategy, and the inclusion and exclusion criteria); (4) satisfy a clear assessment of validity of the findings (e.g., assessment of risk of bias); (5) satisfy a systematic presentation and synthesis of findings beyond those provided by single studies; and (6) reviews of pain assessment tools that include other populations such as premature neonates, toddlers, adolescents or adults were also included provided they have included pain assessment tools used for infants.

The current meta-review excluded reviews with unclear design method, reviews limited to neonates and premature neonates, reviews of systematic reviews, and studies published in languages other than English. To account for the complexity with the use of facial expression in premature neonates [18] and the distinct difference in pain expression in premature neonates and full-term neonates less than 1 month [19, 20], we decided to exclude pain assessment tools exclusively used for this population.

### *Datasourcesandsearchstrategy*

#### *Articleselection*

We searched Scopus, Medline, Embase, CINAHL, MIDRIS, EMCare and Google Scholar (Supplementary Table 2 details databases searched, dates of searches and outcomes, Supplementary Table 3 details the keywords used in the search and Supplementary Table 4 search strategies and outputs for each database searched). EndNote was used to keep the results of the searches of each database and then they were entered into Rayyan, a web-based tool for systematic reviews [21]. After removal of duplicates, all abstracts were initially screened against eligibility criteria by two reviewers in a blinded standardized manner (DA and MA), and then all selected articles were read in full text to receive the final inclusion. Any disagreement between the two reviewers regarding the eligibility of any studies was resolved through discussion and inclusion of a third reviewer (EM).

#### *Dataextractionandanalysis*

For each review included, we extracted descriptive information on study characteristics and study results using a structured data table. Information which was obtained included study authors and year of publication, country, type of review, name of pain assessment tool recommended for infants and type of psychometric data obtained. A list of major definitions of psychometric properties used for extracting data is presented in Table 1. The latter domain also contained the JBI Risk of Bias Assessment Tool for evaluation of study quality. Two reviewers independently extracted data and rated the risk of bias for each of the included studies (DA, MA) and resolved discrepancies through team discussion. Then, the results extracted from the reviews were synthesized using a narrative synthesis approach. Data synthesis was conducted separately for each of the eligible pain assessment tools listed in the review, including general recommendations for using the tool for infants and its psychometric properties. The data extracted for this section was in accordance with the definitions given by the authors in their review and their evaluation of type of measurement property obtained.

**Table 1:** Definitions of psychometric properties and subcategories. (All reliability and validity definitions were extracted and adopted from Walsh & Betz [22]

**Property**	**Definition**
**Reliabilityassessment**	It is the degree to which the measuring of an attribute by a tool is systematic and therefore repeatable.
Internal consistency	It is the degree to which each item of a tool is measuring the same thing as each other item (Cronbach’s alpha, α ≥ 0.70 for group comparisons)
Inter-rater reliability	It is the correlation between ratings of the same rater at two different times, or the product moment correlation between ratings of two different ratters using the same tool (Intraclass correlation coefficient ICC recommended or Pearson’s r ≥ 0.70 for group comparisons, Fleiss' Kapp, or percent agreement)
**Validityassessment**	It is the extent to which the tool actually measures the characteristic or dimension that it is intended to measure.
Face validity	It is the degree to which the content of a test appears relevant to the concept that the tool is intended to measure (judged by a group of experts)
Content validity	It is the degree to which an assessment tool holds an appropriate list of items to represent the concept of interest (basic and minimum index of content validity).
Convergent validity	It is the evidence that different assessment tools developed to measure the same concept all measure the same trait (Pearson’s recommended ≥ 0.40)
Discriminant validity	It is the degree of dissimilarity or distinctness of a tool scores that theoretically represent different trait
Cross cultural validity	It is the degree to which the performance of the assessment tool when being translated or culturally adapted for another population or settings is an adequate reflection of the psychometric performance of the original tool.
Criterion related validity	It is the ability to test if an assessment tool is able to predict a variable that is designated as a criterion or not. It is often measuring the correlation of the instrument with a “gold standard” criterion administered at the same time. It includes two forms (1) predictive validity and (2) Concurrent validity
Construct validity	It is the extent to which a tool measures the construct it is designed to measure and in the settings that it is designed for (factor analysis including exploratory and confirmatory factor analysis)
**Clinicalutility**	It is the degree to which actual use of the assessment tool in clinical settings is associated with changing health outcomes
**Clinicalfeasibility**	It is the practical extent to which an assessment tool can be plausible in a given population or clinical setting

## Risk of bias and methodological quality

The 10 reviews that met our inclusion criteria were assessed for methodological quality by two reviewers independently (DA, MA) using the JBI Critical Appraisal Checklist for Systematic Reviews and Research Syntheses [23]. Disagreements between the two reviewers were resolved through discussion and if needed involvement by a third reviewer (EM).

Quality assessment according to the JBI checklist tool involved 11 assessment criteria: clarity and explicitly of the review questions, inclusion criteria, search strategy, adequacy of sources and resources used, criteria for study appraisal, number of reviewers (2 or more), methods to minimize errors in data extraction, methods used for combined studies, assessment of publication bias, recommendation for policy and practice, and direction for new research (Table 2). Every criterion in the checklist was given a rating of ‘yes’, ‘no’, ‘unclear’ or ‘not applicable,’ and one point was given to every criterion rated ‘yes.’ Using the JBI checklist tool, methodological quality can be judged in terms of “low” if they failed to reach a score of > 50% on critical appraisal, the predetermined cut off score agreed upon by the research team.

**Table 2:** Critical appraisal studies of reviews

**Author(year)**	**Q.1**	**Q.2**	**Q.3**	**Q.4**	**Q.5**	**Q.6**	**Q.7**	**Q.8**	**Q.9**	**Q.10**	**Q.11**	**Totalscore**
**Chan,etal.(2022)** [24]	Y	Y	Y	Y	Y	Y	Y	UN	N	Y	Y	9/11
**Bai&Jiang(2015) **[25]	Y	Y	Y	Y	Y	Y	Y	UN	N	Y	UN	8/11
**Crellinetal.(2007)** [26]	Y	Y	Y	Y	NA	N	N	N	N	Y	Y	6/11
**Crellinetal.(2015)** [27]	Y	Y	Y	Y	Y	Y	Y	NA	N	Y	Y	9/11
**Crellinetal.(2018)** [28]	Y	Y	Y	Y	Y	Y	Y	NA	Y	Y	Y	10/11
**Crostaetal.(2014)** [29]	Y	Y	Y	Y	UN	N	N	UN	N	Y	UN	5/11
**Duhn&Medves(2004)** [30]	Y	Y	Y	Y	N	N	N	N	N	Y	Y	6/11
**Giordanoetal.(2019)** [31]	Y	Y	Y	Y	Y	Y	Y	NA	Y	Y	Y	10/11
**Stapelkampetal.(2011)** [32]	Y	Y	Y	Y	Y	Y	Y	NA	N	Y	Y	9/11
**Maaskantetal.(2016)** [33]	Y	Y	Y	Y	Y	Y	Y	NA	Y	Y	Y	10/11
**Kingsnorthetal.(2015)** [34]	Y	Y	Y	Y	Y	Y	Y	UN	N	Y	Y	9/11

Q.1 Questions are clearly and explicitly stated; Q.2 Inclusion criteria are appropriate for the review question; Q.3 The search strategy is appropriate; Q.4 The sources and resources used to search for studies are adequate; Q.5 The criteria for appraising studies are appropriate; Q.6 Critical appraisal was conducted by two or more reviewers independently; Q.7 There were methods to minimize errors in data extraction; Q.8 The methods used to combine studies are appropriate; Q.9 The likelihood of publication bias was assessed; Q.10 Recommendations for policy and/or practice are supported by the reported data; Q.11 The specific directives for new research are appropriate

*Y* Yes, *N* No, *NA* Not applicable, *UN* Unclear

## Results

### *Selectionofarticles*

**Fig. 1:**
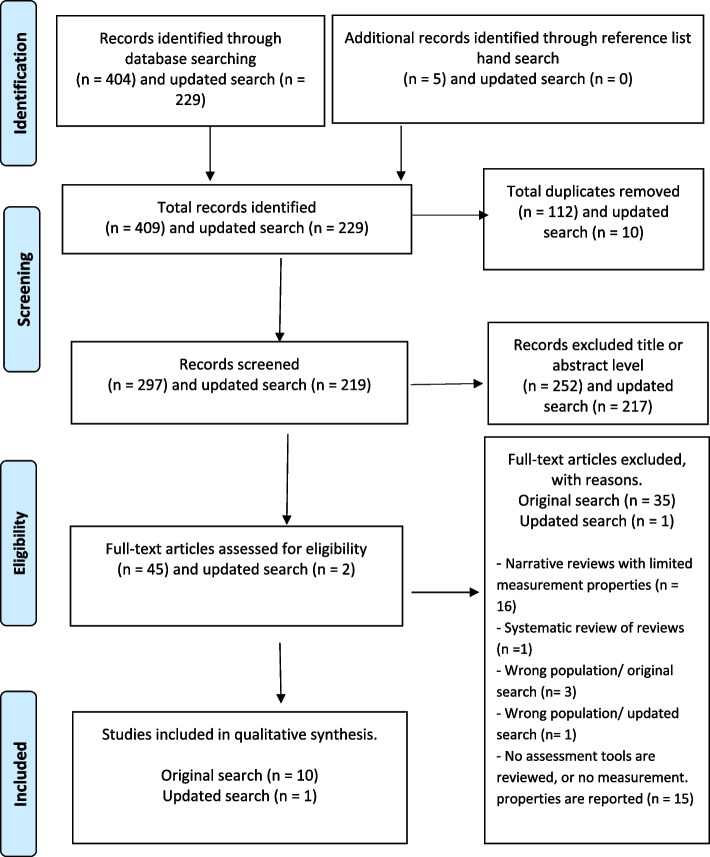
PRISMA flow diagram of records identified in the search of eligible reviews

The literature search produced 409 potentially relevant reviews for screening of which 112 were duplicates, leaving 297 unique reviews. After title and abstract screening 45 were included in the full text review. After full text review, 35 studies were excluded (See Supplementary Table 5), providing a total of 10 studies (See Supplementary Table 6) for data extraction (Figure 1). An updated database search was conducted on the 1^st^ of April 2023 to identify the latest research on the topic. The search yielded 229 studies (Addendum 1). These studies were screened for eligibility as presented in Figure 1. Only one of the studies was identified as eligible. Figure 1 below presents a schema for the search process and the outcome of the article selection process.

### *Characteristicsofincludedreviews*

This meta-review included one systematic integrative review [30] and 10 systematic reviews [24–29, 31–34]. The number of studies included in each review varied from 6 to 250 studies although the number of included studies in some reviews was unclear or not provided (Table 3). The reviews were published between the years 2004 and 2022 and they reported on between 1 to 29 pain assessment tools which can be used for infants aged 1 to 12 months (Table 3).

**Table 3:** Details of the included reviews and the eligible pain assessment tools they included

**Author** **(year)** **Country**	**Review** **type**	**Aim** **of** **the** **review**	**Total** **number** **of** **studies** **included**	**Pain** **assessment** **tools** **reviewed** **related** **to** **infants**	**Ages** **included**	**Setting**	**Risk** **of** **bias**
**Chan,** **et** **al.** **(2022)** [24] **China/** **United** **Kingdom**	Systematic review	To identify pain assessment tools used in palliative care of children	34	DOLIS	0-11 mo	Procedural in cancer	The COSMIN checklist
				COMFORT	0 – 3 yr	Post-operative	
				DEGR	0 – 3 yr	Acute pain	
				MIPS	1 – 7 mo	Post-operative	
				PPP	1 – 18 yr	Neurological and cognitive impairment	
**Bai** **&** **Jiang** **(2015) **[25] **United** **States** **of** **America/China**	Systematic review	To assess the psychometric properties of available assessment tools for measuring pain in Chinese children.	6	FLACC	0-7 yr	Post-operative, Procedural in burn	Psychometric property coding system- CODE
				COMFORT-B	0-7 yr	Post-operative,	
				POCIS	0-5 yr	Procedural pain	
**Crellin,** **et** **al.** **(2007)** [26] **Australia**	Systematic review	To identify and critically analyze the validity and reliability of preverbal and early verbal behavioral pain assessment tools.	N/A	CHEOPS	6-12 mo	Post-operative	Not described
				FLACC	< 3 yr	Post-operative	
				TPPPS	1-5 yr	Post-operative, Acute pain	
				PEPPS	12-24 mo	Post-operative	
**Crellin,** **et** **al.** **(2015)** [27] **Australia**	Systematic review	To evaluate the psychometric properties and utility of the FLACC scale for use in research and to evaluate its appropriateness for clinical and research settings	77	FLACC	6 mo – 4 yr	Post-operative, Procedural/immunization pain, PICU	The COSMIN checklist
**Crellin,** **et** **al.** **(2018)** [28] **Australia**	Systematic review	To assess the research evidence addressing the validity and reliability of the Modified Behavioral Pain scale and its appropriateness for use in children	28	MBPS	0–18 yr	Post-operative, Procedural/immunization pain	The COSMIN checklist
**Crosta,** **et** **al.** **(2014)** [29] **United** **States** **of** **America**	Systematic review	The aims of this review were To evaluate the best available evidence for use of pain assessment tools in hospitalized children with cognitive impairment and to examine their clinical utility in acute care settings.	45 studies	PPP	1–18 yr,	Multi-setting; home, postop, Inpatient. Nonverbal children with cognitive impairment	Not described
**Duhn** **&** **Medves.** **(2004)** [30] **Canada**	Systematic integrative review	To examine the validity and reliability of published pain assessment tools in infants’ tools and to evaluate their reported clinical utility and feasibility	N/A Total of 35 infant pain assessment tools	PRS	1–12 mo	Not described	Not described
				RIPS	Infants & children	Post-operative	
				MAX	0–2 yr	Not described	
				CHEOPS	1 -5 yr	Post-operative	
				CSS or POPS	1–7 mo	Post-operative	
				BPS	3–36 mo	Ventilated child	
				MBPS	4–6 mo	Not described	
				CHIPPS	Newborns, infants & young children	Not described	
**Giordano,** **et** **al.** **(2019)** [31] **Austria**	Systematic review	To provide clinicians with a complete overview on the validity and reliability of the existing pain and sedation scales for different target populations (preterm infants, term infants, and toddlers) and in different clinical contexts.	89 studies	AHTPS	0-16 yr	Acute pain	The COSMIN checklist
				BOPS	1-7 yr	Post-operative	
				CAAS	0-16 yr	Post-operative, Intubated, Ventilated patient	
				CHEOPS	1-5 yr	Post-operative	
				CHIPPS	0-5 yr	Post-operative	
				COMFORT	0-17 yr	Post-operative	
				COMFORT-B	0-3 yr	Pre-post-surgery	
				EVENDOL	0 -7 yr	Acute and prolonged pain	
				FLACC	1 d–7 yr	Acute pain, Post-operative	
				FPS-R	0-3 mo	Post-operative	
				Hartwig Scale	0-10 mo	Procedure	
				MAPS	0-31 mo	Post-surgery	
				MBPS	4-6 mo	Procedure	
				MIPS	1-7 mo	Pre-post-operative	
				MBPS-R	2-12 mo	NA	
				NFCS-R	2-12 mo	Procedure	
				NIPS	2-6 mo	Procedure	
				NAPI	0-36 mo	Post-operative	
				ObsVAS	0-4 yr	Post-surgery	
				POCIS	0–4 yr	Burned child, Post-operative, Acute pain	
				P-MIPS	1-7 mo	Pre- Post-surgery	
				POPS	0–36 mo	Post-operative	
				PEPPS	12-24 mo	Post-operative	
				mPEPPS	12-84mo	Procedure	
				RIPS	0 -36 mo	Post-operative	
				RCEMCPS	0–16 yr	Acute pain	
				TPPPS	12-64 mo	Post-operative	
				TVP	0-13 mo	Acute pain	
				UWCH	0–16 yr	Acute pain	
**Kingsnorth,** **et** **al.** **(2015)** [34] **Canada**	Systematic review	To identify and critique pain assessment tools used for measuring chronic pain in children and evaluate its appropriateness for use in children with cerebral palsy.	250	PPP	1–18 yr	Children with cerebral palsy	Rating schema for assessing validity, reliability and clinical utility
**Stapelkamp,** **et** **al.** **(2011)** [32] **United** **Kingdom**	Systematic review	To review and update the best available evidence on pain assessment in children.	89	AHTPS	<3 yr^a^	During triage ED	The SIGN evidence-grading system
				CAAS	<3 yr^a^	PICU post-operative	
				CMPPMS	<3 yr^a^	Post-operative	
				CHEOPS	<3 yr^a^	Post- and peri-operative	
				COMFORT	<3 yr^a^	Post- and peri-operative	
				DCHPT	<3 yr^a^	Post-operative	
				FLACC	<3 yr^a^	Post-operative and peri-procedural	
				NAPI	<3 yr^a^		
				POPS	<3 yr^a^	Post-operative	
				PPP	1-18 yr	All settings	
				PRS	<3 yr^a^	Post-operative	
				TPPPS	<3 yr^a^	Post-operative	
				UWCH	<3 yr^a^	Peri-procedural pain	
				ObsVAS	<3 yr^a^	Post-operative pain	
**Maaskant,** **et** **al.** **2016** [33] **Netherlands**	Systematic review	To evaluate the methodological quality of studies used the COMFORT scale in children up to 18 years.	30	COMFORT	0–18 yr	PICU, Non ventilated neonates, NICU	The COSMIN checklist

*AHTPS* Alder Hey Triage Pain Scale, *BOPS* Behavioural Observational Pain Scale, *BPS* Behavioural Pain Score, *CAAS* Cardiac Analgesic Assessment Scale, *CHEOPS* The Children Hospital of Eastern Ontario Pain Scale, *CHIPPS* Children’s and Infants Postoperative Pain Scale, *CHIPPS or KUSS* Children´s & Infant´s Postoperative Pain Scale, *CSS* Clinical Scoring System, *CMPPMS* Chedoke-McMaster Paediatric Pain Management Sheet, *DCHPT* Derbyshire Children’s Hospital Pain Tool, *DEGR* Douleur Enfant Gustave Roussy Scale, *FLACC* Face, Legs, Activity, Cry and Consolability, *EVENDOL* Evaluation Enfant Douleur, *FPS-R* Faces Pain Scale Revised, *MAPS* Multidimensional Assessment Of Pain Scale, *MAX* Maximally Discriminative Facial Movement Coding System, *MBPS* Modified Behavioural Pain Scale, *MIPS* Modified Infant Pain Scale, *mo* months, *NAPI* Nursing Assessment of Pain Intensity Revised, *NFCSr* Neonatal Facial Coding System-Revised, *NIPS* Neonatal Infant Pain Scale, *ObsVAS* Observer Visual Analog Scale, *PEPPS* Preverbal, Early verbal Pediatric Pain Scale, *P-MIPS* Partial Modified Infant Pain Scale, *POCIS* Pain Observation Scale for Young Children, *POPS* Postoperative Pain Score, *PPP* Paediatric Pain Profile, *PRS* Pain Rating Scale, *RCEMCPS* Royal College of Emergency Medicine Composite Pain Scale, *RIPS* Riley Infant Pain Scale, *TPPPS*: Toddler Pre-schooler Postoperative Pain Score, *TVP* Touch Visual Scale, *UWCH* University of Wisconsin Children’s Hospital Pain Scale, *yr* years

^a^Age < 3 yr excludes neonates

### Synthesis and *findings*

We identified 11 reviews that contained tools measuring pain in infants (1-12 months) and provided psychometrics analysis of 36 tools. Two tools were assessed in five reviews: the FLACC (Face, Legs, Activity, Cry and Consolability) [25–27, 31, 32] and the COMFORT/COMFORT-behaviour [24, 25, 31–34], while two were included in four reviews, namely the CHEOPS (The Children Hospital of Eastern Ontario Pain Scale) [25, 30, 32, 33] and the PPP (Paediatric Pain Profile) [24, 29, 32, 34]. The MBPS/MBPS-R (Modified Behavioral Pain Scale/ Modified Behavioral Pain Scale Revised) [28, 30, 31], the CSS/POPS (Clinical Scoring System also referred to as the Post-Operative Pain Score) [30–32], and the TPPPS (Toddler Pre-schooler Poster-Operative Pain Scale) [26, 31, 32] were included in three reviews. While the CHIPPS (Children´s & Infant´s Postoperative Pain Scale) [29, 31], the PEPP/mPEPP (Preverbal, Early verbal Pediatric Pain Scale/modified Preverbal, Early verbal Pediatric Pain Scale) [26, 31]; and RIPS (Riley Infant Pain Scale) [30, 31] were included in two reviews. The remainder of the tools were assessed only by one review.

### *Settingswherethetoolswerestudied*

The child population varied across reviews and some reviews included mixed population of newborns and infants [30, 32, 33] infants and toddlers [30, 32], infants and older children or adolescents [24, 27, 28, 31, 32, 34] . Two reviews were limited to children with cognitive impairments [29], or cerebral palsy [34]. Twenty-two tools focused on pre-, peri- and/or post-operative pain, 10 tools focused on procedural pain and 10 on acute pain, while the rest were not adequately described. The terms used to describe the use in hospital settings included bedside, Paeditaric Intensive Care Unit (PICU), intensive care, ventilated child, sedated child, post analgesic, and pre-operative, peri-operative and post-operative. The terms used to describe the use for acute procedural settings also varied and included procedural/ immunization/ outpatient clinic, triage in Accident & Emergency or procedural outpatient.

### *Assessmentofevidenceofmeasurementpropertiesofthetools*

We described the detailed study characteristics in Table 3 and the measurement properties in Table 4. The quality of retrieved reviews was assessed at the level of data synthesis, concerning the quality of methodology. Five reviews used the Consensus-based Standards for the selection of health Measurement Instruments (COSMIN) checklist [24, 27, 28, 31, 33] one review used the Psychometric Property Coding System – CODE [25] and one review used the Scottish Intercollegiate Guidelines Network (SIGN evidence-grading system) [32] . The authors of one review critiqued tools on the merit of their validity and reliability using the rating schema for assessing clinical utility developed by the Society of Paediatric Psychology Assessment Task Force [34], while the rest of retrieved reviews did not describe their rating system [26, 29, 30]. It is worth noting here that there are differences between the assessments of evidence of measurement properties used between studies. For example, COSMIN [35] is used to evaluate measurement properties of a scale (scale level), and SIGN [36] is used to evaluate the overall methodological quality of primary measurement studies. On the other hand, the original psychometric properties coding system for evaluating methodological quality of the assessment scales developed by Zwakhalen et al. [37] was adopted and refined for use by Bai and Jiang [25]. The differences between the reviews in terms of their overall aim, inclusion criteria, and their rating system made it difficult to aggregate the results and make a conclusion about the psychometric properties of the tools included in this review.

**Table 4:** Measurement properties of pain assessment tools for infants extracted included reviews

Author (year)	Assessment tool	Reliability assessment			Validity assessment	
		**Internal** **consistency**	**Inter-rater** **reliability**	**Intra-rater** **reliability**	**Face/** **Content** **validity**	**Convergent** **validity**
**Chan,** **et** **al.** **(2022)** [24]^**a**^	COMFORT	High	High	-	Moderate	-
	DOLLS	High	Moderate	-	-	
	FLACC	Moderate	High	-	-	-
	MIPS	-	Very low	-	-	-
	PPP	High	Moderate	-	Very low	-
**Bai** **&** **Jiang.** **(2015) **[25]^**b**^	FLACC	α = 0.75 – 0.799	ICC = 0.79 – 0.84 r = 0.65 – 0.95	-	Yes	Acceptable to high
	COMFORT- behavior	α = 0.869 – 0.83	ICC = 0.82 – 0.9	-	Yes	-
	POCIS	α = 0.846 – 0.856	ICC = 0.78 – 0.80, r = 0.66	-	Yes	-
**Crellin,** **et** **al.** **(2007)** [26]^**a**^	CHEOPS	Moderate to good	Very good		-	-
	FLACC	-	Good	-	-	Demonstrated
	TPPS	Poor to Good	Good	-	-	-
	PEPPS	Poor to good	Very good	Very good	-	
**Crellin,** **et** **al.** **(2015)** [27]^**a**^	FLACC	Moderate	Low, Moderate, High	Moderate to High	Low	Very low to moderate
**Crellin,** **et** **al.** **(2018)** [28]^**a**^	MBPS	Low to Moderate	Low, Moderate, High	Moderate to High	-	Nil to Low
**Crosta,** **et** **al.** **(2014) **[29] ^**(d)**^	PPP	α = 0.75 – 0.89	ICC = 0.70 – 0.89	-	Yes	-
**Duhn** **&** **Medves.** **(2004)** [30]^**a**^	PRS	-	*r* = 0.65 to 0.84, *P* = 0.0001	-	-	-
	RIPS	α = 0.87 to 0.93	ICC = 0.39–0.87	-	-	-
	MAX	-	Agreement83%	-	-	-
	CHEOPS	-	Agreement90 to 99.5%	-	Yes	r = 0.85, *p* < 0.05
	CSS or POPS	-	-	-	-	-
	BPS	-	-	-	-	-
	MBPS	-	ICC = 0.95, *p* < 0.001	-	-	-
	CHIPPS	α = 0.96	r = 0.93	-	-	-
**Giordano** **et** **al.** **(2019)** [31]^**a**^	AHTPS	-	Demonstrated	-	Yes	-
	CAAS	-	Demonstrated	-	Yes	-
	BOPS	-	Demonstrated	-	Yes	
	CHEOPS	Demonstrated	Demonstrated	-	Yes	Established (with VAS)
	CHIPPS	Demonstrated	Demonstrated	-	Yes	-
	COMFORT	Demonstrated	Demonstrated	-	Yes	-
	COMFORT-B	-	Demonstrated	-	Yes	-
	EVENDOL	-	Demonstrated	Demonstrated	Yes	-
	FLACC	Demonstrated	Demonstrated	Demonstrated	Yes	-
	FPS-R	-	Demonstrated	-	Yes	-
	Hartwig	Demonstrated	Demonstrated	-	Yes	Established
	MAPS	Demonstrated	-	-	Yes	-
	MBPS	Demonstrated	Demonstrated	Demonstrated	Yes	-
	MBPSr	Demonstrated	-	Demonstrated	Yes	-
	MIPS	-	-	-	Yes	-
	NFCSr	Demonstrated	-	-	Yes	-
	NIPS	Demonstrated	Demonstrated	-	Yes	-
	NAPI	Demonstrated	Demonstrated	-	Yes	-
	ObsVAS	-	Demonstrated	-	Yes	-
	POCIS	Demonstrated	Demonstrated	-	Yes	-
	PMIPS	-	-	Demonstrated	Yes	-
	POPS	-	-	-	Yes	-
	PEPPS	Demonstrated	Demonstrated	Demonstrated	Yes	-
	mPEPPS	Demonstrated	Demonstrated	-	Yes	-
	RIPS	-	Demonstrated	Demonstrated	Yes	-
	RCEMCPS	-		Demonstrated	Yes	-
	TPPPS	Demonstrated	Demonstrated	-	Yes	
	TVP	-			Yes	-
	UWCH	-	Demonstrated	Demonstrated	Yes	-
**Kingsnorth** **et** **al.** **(2015)** [34]^**f**^	PPP	Well-established				
**Stapelkamp** **et** **al.** **(2011)** [32]^**c**^	AHTPS	-	Acceptable	-	-	
	CASS	-	Acceptable	-	-	-
	CMPPMS	-	Acceptable	-	-	-
	CHEOPS	-	Acceptable	-	-	-
	COMFORT	-	Acceptable	-	-	-
	DCHPT	-	Acceptable	-	-	-
	FLACC	-	Acceptable	-	-	-
	NAPI	-	Acceptable	-	-	-
	POPS	-	Acceptable	-	-	-
	PPP	-	Acceptable	-	-	-
	PRS	-	Acceptable	-	-	-
	TPPPS	-	Acceptable	-	-	-
	UWCH	-	Acceptable	-	-	-
	ObsVAS	-	Acceptable	-	-	-
**Maaskant** **et** **al.** **(2016)** [33] ^**a**^	COMFORT	Poor, Fair, Good, and excellent*	Poor, Fair, Good, and excellent*	-	Yes	-

Author (year)	Validity assessment				Clinical utility/ feasibility
	**Discriminant** **validity**	**Cross-cultural** **validity**	**Criterion/** **concurrent** **validity**	**Construct** **/** **Structural/** **validity**	
**Chan,** **et** **al.** **(2022)** [24]^**a**^	-	-	Low	Moderate	Low responsiveness
		-	High	-	Moderate responsiveness
	-	-	Low	-	Moderate responsiveness
	-	-	Very low	-	-
	-	High	Moderate	-	Low responsiveness
**Bai** **&** **Jiang.** **(2015) **[25]^**b**^	Good	Supported	Supported	Acceptable to high	Very good feasibility
	-	Supported	-	Acceptable to high	Very good feasibility
	-	Supported	Supported	Acceptable to high	Very good feasibility
**Crellin,** **et** **al.** **(2007)** [26]^**a**^	-	-	Moderate to strong (r = 0.743–0.921)	Weak to good	Burdensome to apply
	Demonstrated	-	Good	Supported	-
	Demonstrated	-	-	Good	-
	-	-	-	Demonstrated	-
**Crellin,** **et** **al.** **(2015)** [27]^**a**^	Low	Supported	Low	-	Low to moderate feasibility and utility
**Crellin,** **et** **al.** **(2018)** [28]^**a**^	Nil, Low, Moderate, High	Not reported	Nil to Low	-	Insufficient evidence of feasibility or clinical utility
**Crosta,** **et** **al.** **(2014) **[29] ^**(d)**^	-	-	Supported	Supported	Limited feasibility and clinical utility
**Duhn** **&** **Medves.** **(2004)** [30]^**a**^	Established	-	-	-	None reported
	Established	-	-	-	None reported
	-	-	-	-	Low
	-	-	-	Supported (r = 0.85, *P* < 0.05))	Good
	Demonstrated	-	-		Limited
	Weak	-	-	-	None reported
		-	Established (With VAS) r = 0.68 and 0.74. *p* < 0.001	Established	None reported
	-	-	Good, (with TPPPS) Agreement 87.4%	-	Easy to use
**Giordano** **et** **al.** **(2019)** [31]^**a**^	-	-	Established (with WBFPS)	Established	None reported
	-	-	Established (with VAS)	Established	
			Established (with CHEOPS)	Established	
	-	-	-	Established	
	-	-	Established (with TPPPS)	Established	
	Yes	-	Established (with VAS)	Established	
	-	-	Established (with CASS)	Established	
	-	-	Established (with EDIN, FLACC, TPPPS, CHEOPS)	-	
	-	-	Established (with MBPS, NIPS, OPS)	established	
	-	-	Established (with PASS)	-	
	-	-	Established (with COMFORT, VAS)	-	
	-	-	Established (with FLACC, VAS)	Established	
	-	-	Established (with, VAS)	Established	
	-	-	Established (with, NFCSr)	Established	
	-	-	-	Established	
	-	-	Established (with, MBPSr)	Established	
	-	-	Established (with, FLACC, NIPS)	-	
	-	-	-	Established	
	-	-	Established (with, MAPS, NIPS)	-	
	-	-	-	Established	
	-	-	-	Established	
	-	-	-	Established	
	-	-	-	Established	
	-	-	-	Established	
	-	-		Established	
	-	-			
			Established (with VAS)	Established	
	-	-	Established (with NRS)		
	-	-	Established (with BWFS)	Established	
**Kingsnorth** **et** **al.** **(2015)** [34]^**f**^	Well-established				Moderate clinical utility
**Stapelkamp** **et** **al.** **(2011)** [32]^**c**^	-	-		Demonstrated	High usability, Moderate clinical utility
	-	-	-	Demonstrated	
	-	-	-	Demonstrated	
	-	-	-	Demonstrated	
	-	-	-	Demonstrated	
	-	-	-	Demonstrated	
	-	-	-	Demonstrated	
	-	-	-	Demonstrated	
	-	-	-	Demonstrated	
	-	-	-	Demonstrated	
	-	-	-	Demonstrated	
	-	-	-	Demonstrated	
	-	-	-	Demonstrated	
	-	-	-	Demonstrated	
**Maaskant** **et** **al.** **(2016)** [33] ^**a**^	Demonstrated	-	-	Poor, Fair and Good*	Not reported, Fair, Good, and Excellent for responsiveness

Acceptable (Inter-rater reliability) = r ≥ 0.60

*α* Cronbach alpha, *K* Kappa, *ICC* Intraclass Correlation Coefficient, *r* correlation coefficient (Pearson’s r), *CVI* Content Validity Index, - No information provided, *AHTPS* Alder Hey Triage Pain Scale, *BOPS* Behavioural Observational Pain Scale, *BPS* Behavioural Pain Score, *CAAS* Cardiac Analgesic Assessment Scale, *CHEOPS* The Children Hospital of Eastern Ontario Pain Scale, *CHIPPS* Children’s and Infants Postoperative Pain Scale, *CHIPPS or KUSS* Children´s & Infant´s Postoperative Pain Scale, *CSS* Clinical Scoring System, *CMPPMS* Chedoke-McMaster Paediatric Pain Management Sheet, *DEGR* Douleur Enfant Gustave Roussy Scale, *DCHPT* Derbyshire Children’s Hospital Pain Tool, *FLACC* Face, Legs, Activity, Cry and Consolability, *EVENDOL* Evaluation Enfant Douleur, *FPS-R* Faces Pain Scale Revised, *MAPS* Multidimensional Assessment Of Pain Scale, *MAX* Maximally Discriminative Facial Movement Coding System, *MBPS* Modified Behavioural Pain Scale, *MIPS* Modified Infant Pain Scale, *mo* Months, *NAPI* Nursing Assessment of Pain Intensity Revised, *NFCSr* Neonatal Facial Coding System-Revised, *NIPS* Neonatal Infant Pain Scale, *ObsVAS* Observer Visual Analog Scale, *PEPPS* Preverbal, Early verbal Pediatric Pain Scale, *P-MIPS* Partial Modified Infant Pain Scale, *POCIS* Pain Observation Scale for Young Children, *POPS* Postoperative Pain Score, *PPP* Paediatric Pain Profile, *PRS* Pain Rating Scale, *RCEMCPS* Royal College of Emergency Medicine Composite Pain Scale, *RIPS* Riley Infant Pain Scale, *TPPPS* Toddler Pre-schooler Postoperative Pain Score, *TVP* Touch Visual Scale; UWCH: University of Wisconsin Children’s Hospital Pain Scal

^a^Rating based on the COSMIN checklist quality

^b^Rating based on the Psychometric property coding system- CODE

^c^Rating based on the SIGN-grading system

^d^Rating system not described

^e^Rating based on Risk of bias_ Combined ROBIS domains

^f^Rating system not described, except for overall evaluation of V&R and clinical utility using rating schema for assessing clinical utility

^*^statistically significant difference

### *Psychometricdataofthepainassessmenttoolsininfant*

The 36 pain assessment tools were assessed for properties such as internal consistency (18 tools), content/face validity (30 tools), criterion validity (23 tools), construct validity (23 tools), and cross-cultural validity (3 tools). Overall, and as shown in Table 4, the methodological quality reported in the reviews varied significantly and ranged from ‘acceptable’ for the POCIS [25] to very good’ for the FLACC, COMFORT-Behavior (COMFORT-B), and POCIS [25] , and from high risk of bias for the Nursing Assessment of Pain Intensity Revised (NAPI), POCIS, Post-Operative Pain Score (POPS), Riley Infant Pain Scale (RIPS), and University of Wisconsin Children’s Hospital Pain Scale (UWCH) to low risk of bias for the EValuation ENfant DOuLeur (EVENDOL) [31]. For the MBPS, inconsistent level of evidence was reported [28].

### Reliability

Reliability assessment of the pain tools was evaluated using internal consistency, inter-rater and/or intra-rater reliability. There were no reliability data reported for three of the tools, namely the Behavioral Pain Score (BPS), Partial Modified Infant Pain Scale (MIPS), and Touch Visual Pain scale (TVP). Several reviews reported that tools had internal consistency or inter/intra-rater reliability without providing details on type of reliability data obtained for the tool [24, 31, 32, 34]. Although, in the case of Stapelkamp et al. [32] acceptable inter-rater reliability (correlation ≥ 0.6) was used as an inclusion criterion for observer-rated tools, without additional data being provided for individual tools in the review.

In six reviews the authors presented an evaluation of the methodological quality of the studies used to evidence the reliability of a range of tools including the FLACC, Toddler Pre-schooler Postoperative Pain Score (TPPPS), Preverbal, Early verbal Pediatric Pain Scale (PEPPS), MBPS, COMFORT-B [24, 26–28, 33]. The quality of the studies varied considerably from poor, poor/fair to good/excellent. Issues around the sample size, lack of blinding, assessments using comparator tools completed by the same person, and the comparator not being an acceptable standard were often cited as reasons for quality issues in the primary studies. When the evaluation of quality was applied to the findings of the primary studies to derive the level of evidence of reliability this too varied from low to moderate to high for the FLACC [27] and MBPS [28].

Studies used Cronbach alpha for reports of internal consistency, whereas kappa coefficients, percentage agreement, correlation coefficients, and intra-class correlation coefficients were used to describe inter and intra-rater reliability. The variation in methods used for calculation of reliability and reports made direct comparisons difficult.

### Validity

Validity assessment of the pain tools was reported using indicators of face/content validity, convergent validity, discriminant validity, criterion/concurrent validity, and/or construct validity. Evidence was provided in four reviews [24–27] on the cross-cultural validity of six assessment tools, namely the FLACC, COMFORT-B, POCIS, CHEOPS, PPP, and TPPPS, when used in a different language or cultural context. Additionally, some reviews reported that tools had ‘established’ or ‘supported’ criterion validity [31] and similarly so for discriminant validity [31, 34] without providing supportive data or their reason for this conclusion. In the case of Stapelkamp et al. [32] observational pain assessment tools were included if there was “both demonstrated known groups validity and inter-rater reliability).” In this case, demonstration of construct validity (i.e., the ability to differentiate between pain and non-pain states) was seen as more important than cross-validation of tools with others (criterion validity) in the absence of a gold standard. Data used to support the various tools construct validity was not provided within the review itself.

Studies evaluated validity of the pain assessment tools mostly reported ‘acceptable’ to ‘high’ quality [25-28, 33, 34]. One study identified weak or limited validity data for the BPS [30] and there were no validity data reported for two assessment tools, namely the Maximally Discriminative Facial Movement Coding System (MAX), and Royal College of Emergency Medicine Composite Pain Scale (RCEMCPS). Overall, the most common tools used when comparing pain scores of one assessment tool to another (criterion/concurrent validity) were the Observer administered Visual Analogue Scale (ObsVAS), Neonatal Infant Pain Scale (NIPS), FLACC and COMFORT.

### *Clinicalutilityandfeasibility*

Data on the utility, responsiveness and feasibility of pain assessment tools used in infants were very limited. Only eight tools (FLACC, COMFORT, POCIS, MAX, CSS, Children´s & Infant´s Postoperative Pain Scale (CHIPPS, CHEOPS, and PPP) were reported to have some evidence of utility and/or feasibility. Specific evaluation for feasibility appears to have been carried out only for three tools, namely the FLACC[24, 25], COMFORT[24, 25] and POCIS [25] whereas specific data on clinical utility have been undertaken for only one tool namely: the PPP [24, 29, 32, 34] with a limited focus for use in children with cognitive impairment.

An instance of conflicting data on utility and feasibility of the FLACC from two different reviews [25, 27] was attributed mainly to heterogeneity of studies, population and settings used to evaluate feasibility of the FLACC and therefore make it difficult to confidently draw broad conclusions on the tool’s feasibility and/or utility. It must be highlighted that when Bai and Jiang [25] assessed feasibility of the FLACC, COMFORT and POCIS, the main aspects assessed by the reviewers were the short length of items and clear user instructions. On the other hand, and for the PPP, earlier reviews clearly indicated that claims of tool feasibility and/or utility were based on time required to administer the tools [24, 29, 34] and/or brevity of a tool to its usefulness [24, 29, 32, 34]. Additional dimensions of clinical utility assessed by Kingsnorth et al.[34] included comprehensiveness, such as impact of pain, consideration of varied gross motor abilities, and considerations of varied verbal abilities of the child. For the PPP, another review suggested additional specifications for the tool suitability, such as age and cognitive abilities of the child, clinical setting, and the need for additional training to use by the health care practitioners [32].

The need for additional training/teaching in the use of the PPP was also judged as important by Crosta et al. [29], mainly with relation to the need for better instructions on scoring of the 20 items of the PPP to add clarity and help clinicians in the interpretation of the score’s meaning. For the PPP it was stated that authors of studies/tools did not report on what degree they found the PPP easy to use, or if it was time-consuming and complex for assessment and documentation, or the degree of friendliness for both child and family [32]. There were four tools which were described as ‘easy to use’ by multiple reviews, namely the CHEOPS, CHIPPS, CSS, and MAX, without providing further supporting evidence [30]. Crellin et al.[28] suggested there was insufficient data to evaluate the feasibility or utility of the MBPS.

## Discussion

Eccleston et al. [7] have set making pain visible as one of their translational goals to improve pediatric pain management. To achieve this there is a need for valid and reliable observational pain assessment tools which inform clinical decision making and can be easily incorporated into clinical practice. Therefore, there is a need for evidence which supports the psychometric properties (validity and reliability) and feasibility as well as clinical utility of such tools, which is what this meta-review aimed to synthesize. What was demonstrated is that there are over 36 pain assessment tools which can been used to evaluate pain in infants aged 1 to 12 months. However, based on currently available data, no single tool can be recommended as the “gold standard”. This confirms the need for further research and development with the focus on arriving at a pain assessment tool which has sound psychometric and clinical utility properties. For our study we adopted the NICE definition as a gold standard (https://www.nice.org.uk/glossary?letter=g): “A method, procedure or measurement that is widely accepted as being the best available to test for or treat a disease”. In our case to achieve the status of gold standard it had to be considered the best available tool to measure pain in terms of reliability, validity, feasibility and usability.

Only one of the 11 reviews included in the current meta-review focused specifically on infants [30], the remainder included across a range of aged groups, such as premature and full-term neonates, toddlers, young children, and adolescents. From these reviews we extracted and analyzed data for 36 tools. The majority of the tools were multidimensional (exceptions being the NFCS-R, FPS-R, MAX and the ObVAS) and varied in their derivation, content, and rating of the pain scores. Many of the tools have been applied to children who are intubated, sedated and ventilated, including infants. Further, they have been used for acute pain, pre-, peri- and post-operative pain, procedural pain, including that associated with immunization, and chronic pain. An important lesson learnt that pain assessment tools in infants were mostly focused on acute pain, whereas chronic pain was relatively ignored. Of the tools identified in this review, the COMFORT scale was the main tool that can differentiate stages of pain and assess pain temporality. However, traditional definition of chronic pain may not apply in infant population. Therefore, our discussion in this regard has been hampered by difficulties in rectifying differences between acute and persistent/prolonged pain in definitions, as well as in their clinical picture. The fact that infants have not lived enough to meet chronic pain criteria applied to older children, and that most studies were designed to measure acute pain in predictable situations, such as painful procedures, may have contributed to the researcher’s inability to predict or adequately measure chronic pain [38]. The use of explicit pain definitions and the integration of biological and behavioral/observational evidence could ultimately inform a more accurate assessment of infants who suffer from prolonged and persistent pain and assist in pain management.

The most cited tools in the included reviews were the FLACC, COMFORT, CHEOPS, followed by the MBPS, POPS, TPPPS and PPP. The findings of this meta-review in terms of the most commonly cited tools were in line with those identified by an earlier meta-review by Andersen et al. [11]. They reported that the previous systematic reviews had most frequently recommended the FLACC, COMFORT, and CHEOPS. Yet differences between the reviews in terms of their inclusion criteria, rating system and incomplete data synthesis made it impossible to recommend one particular tool to use. Given the variety of clinical settings in which the tools may be used and the different etiologies of pain this may not be unexpected. However, further evidence of the psychometric properties and clinical application and utility of these tools in practice would allow such recommendation to be made. Routine application of the COSMIN “Guideline for selecting outcome measurement instruments for outcomes included in a Core Outcome Set” [39] in the evaluation of current and future pain assessment tools would allow potential aggregating of data, thus strengthening the evidence pool.

This meta-review found that measures of inter-rater reliability, face/content validity and construct validity are the types of measurement properties most commonly and comprehensively reported, whereas information on clinical utility and cross-cultural validity is seldom and poorly reported. While it may be argued that certain aspects of validity and reliability are more important than others, we believe that evidence should be looked at as a whole. To date, there is no hierarchy of evidence described for the different psychometric properties described by earlier studies. Therefore, the collective opinion of earlier studies is regarded as the best available source of advice on offering an evaluation about what constitutes a validated and a reliable tool. Nevertheless, while many pain scales have focused on validity related to inter-rater and intra-rater reproducibility, it could be argued that looking at more scale properties, such as responsiveness to a known treatment may be more fundamental.

As suggested by Duhn & Medves [30] further evaluation of clinical utility and feasibility of existing pain assessment tools is needed to enhance our ability to accurately assess pain in the infant population. Furthermore, evaluation of clinical utility and feasibility would provide important information in regard to the appropriateness of existing tools across different clinical settings. As suggested by Eccleston et al. [7] in clinical practice pain in infants tends to be both underdiagnosed and undertreated by clinicians. This is compound by the lack of a gold standard tool used to measure pain in infants. Certainly, a gold standard cannot be achieved without the tool having a clearly evidenced clinical utility and feasibility, in addition to sound psychometric properties.

Studies focused on tools’ clinical utility and feasibility generally have not moved forward in a systematic nor rapid way because of the logistical problems connected with use of pain assessment tools in vulnerable populations. It could also be related to the lack of clarity about what constitutes clinical utility in the literature and how it can be evaluated [40]. The length of items used in pain assessment tools, ease of use, time needed to complete/ document the assessment, and clear user instructions are examples of aspects that have the potential to influence both feasibility of use and clinical utility in clinical practice. Therefore, these should be reported by studies, in addition to other evaluation of reliability and validity. Additionally, future studies should also consider current conflicting data on utility and feasibility of tools [24, 25, 27, 29, 32, 34] by addressing issues arising from heterogenicity of studies, dimensions evaluated, heterogeneity of populations and settings that makes it difficult to conclusively evaluate clinical utility and feasibility.

It is worth noting that Patient Related Outcomes Measures (PROMs) [39] are critical to evidencing the clinical utility of pain assessment tools. Establishing valid and reliable assessment of pain in clinical practice leads to improved patient outcomes (e.g., less pain, better pain management, better quality of life). Proctor’s conceptual model of implementation science outcomes is another approach that could be used in future studies to present evidence of the feasibility of use of current pain assessment tools in infants and related outcomes [41]. The described implementation science methodology and the PROMs could be used as a framework for future studies.

We noted also that nine of the 11 reviews were conducted in Western countries and cultural validity of the pain assessment tools were only addressed in four; one review by Bai & Jiang [25] with special focus Chinese children and included the FLACC, COMFORT-B and POCIS, and those of Crellin et al. [24, 26–28] which covered the FLACC, TPPPS and MBPS. There are several variations in the way different individuals interpret pain assessment tools as a function of their age, literacy levels, and cultural background [42]. We can argue, therefore, that the appropriateness of using one measure created in one culture or setting for another setting is a matter of concern, especially in developing countries where the social, educational, and economic context differs dramatically from Western countries. Exploration of cross-cultural validity provides suggestive evidence of potential for use within diverse cultural and linguistic context.

Given that there is still no ground truth for the measurement of pain in infants, certain aspects of validity testing are hard to estimate. COSMIN guideline[35] for systematic reviews of patient-reported outcome measures is increasingly seen as measurements that may help overcome of the above-mentioned methodological problems. Future studies can achieve greater rigor and relevance by (i) ensuring that a tool’s ‘responsiveness’ is tested against known analgesics; (ii) testing ‘construct validity’ such as ‘hypothesis testing validity’ and (iii) ensuring that the Importance/Assessment of ‘Interpretability’ is considered when constructing and testing new tools.

Considering difficulties in assessing pain in populations unable to communicate, including infants, parental involvement remains an important component for assessment of pain. Nurses and clinicians often rely on parent’s input to document infant’s behavioral responses, especially when the child is too young to verbalize their pain or symptoms[43]. Inclusion of providers and parental input to identify the early stages of pain is similar to that made previously by an earlier review[38] and is based on pediatric committee consensus [43]. There is also a growing interest in addressing such challenges by leveraging from technological advances. In this regard, a review by Zamzmi et al. [3] showed that the use of automated methods for pain analysis and recognition may be a better fit for pain assessment in infants. Therefore, considering developments in machine learning based techniques for measuring pain in this population group, it can be expected that automation of pain assessment could have a number of implications in clinical practice that will result in a) development of pain assessment tools whose automation is driven by artificial intelligence; b) digitization of pain assessment documentation; c) enabling of rapid point-of-care assessment and d) continuous monitoring of pain. Continuous monitoring and predictive algorithms developed by artificial intelligence (AI) is another approach that may assist in measuring aspects of pain, including chronic pain[42]. These developments may well have a positive impact on addressing the four transformative goals proposed by Eccleston et al. [7] and therefore improve the lives of children experiencing pain as well as their families.

## Strengths and limitations

The methodology approach adopted for this meta-review was a strength as it provided a comprehensive and a practical way of obtaining information on currently used pain assessment tools compared to undertaking reviews of primary studies. Another strength is the rigorous approach adopted in the review process as we attempted to minimize bias by having each review screened independently by at least two reviewers. The main limitation of this study was that identification of all pain assessment tools used in infants could not be guaranteed, as not all of them may have been included in identified systematic reviews. Furthermore, pain assessment tools not published in English may also have been missed. Whilst we checked further available data when readily available, such as in supplementary files, we did not communicate with the authors to obtain missing data. Finally, our search did not specifically target reviews reporting on the feasibility or clinical utility of the pain assessment tools, therefore our evidence may be more limited on these aspects than it might have been otherwise.

## Conclusion

The assessment of pain in infants remains complex and challenging for practitioners and researchers, mainly because infants are unable to self-report their pain. The 11 published reviews included in this meta-review evaluated 36 tools of which seven of these were included at least by three reviews. The level of evidence reported in regard to each tool’s psychometric properties varied widely across published reviews, and only a few of these reviews reported on feasibility and clinical utility and had conflicting results or measured different dimensions of utility. Therefore, we were unable to conclude that any one tool is better than the other without proper caution. To address this there is a need for standardization of the evaluation of psychometric properties of pain assessment tools, together with a greater focus feasibility and clinical utility. Underpinning the latter is the need for accompanying PROMs data, indicating the systematic use of the tool results the best possible pain management outcomes.

## Supplementary Information

**Additionalfile1:SupplementaryTable1.** Selection Criteria for the Systematic Review (PICOS). **SupplementaryTable2.** Databased searched, search dates and outputs . **SupplementaryTable3.** Database search strategies and outputs. **SupplementaryTable4.** List of search terms. **SupplementaryTable5.** List of Excluded reviews. **SupplementaryTable6.** List of included reviews.

## Data Availability

All data generated or analysed during this study are included in this published article [and its supplementary information fles].

## Abbreviations

AHTPS: Alder Hey Triage Pain Scale
BOPS: Behavioural Observational Pain Scale
BPS: Behavioural Pain Score
CAAS: Cardiac Analgesic Assessment Scale
CHEOPS: The Children Hospital of Eastern Ontario Pain Scale
CHIPPS: Children’s and Infants Postoperative Pain Scale
CHIPPS or KUSS: Children´s & Infant´s Postoperative Pain Scale
CSS: Clinical Scoring System
CMPPMS: Chedoke-McMaster Paediatric Pain Management Sheet
DCHPT: Derbyshire Children’s Hospital Pain Tool
FLACC: Face, Legs, Activity, Cry and Consolability
EVENDOL: Evaluation Enfant Douleur FPS-R: Faces Pain Scale Revised
MAPS: Multidimensional Assessment Of Pain Scale
MAX: Maximally Discriminative Facial Movement Coding System
MBPS: Modified Behavioural Pain Scale
MIPS: Modified Infant Pain Scale
Mo: months
NAPI: Nursing Assessment of Pain Intensity Revised
NFCSr: Neonatal Facial Coding System-Revised
NIPS: Neonatal Infant Pain Scale
ObsVAS: Observer Visual Analog Scale
PEPPS: Preverbal, Early verbal Pediatric Pain Scale
P-MIPS: Partial Modified Infant Pain Scale
POCIS: Pain Observation Scale for Young Children
POPS: Postoperative Pain Score
PPP: Paediatric Pain Profile; PRS: Pain Rating Scale
RCEMCPS: Royal College of Emergency Medicine Composite Pain Scale
RIPS: Riley Infant Pain Scale
TPPPS: Toddler Pre-schooler Postoperative Pain Score
TVP: Touch Visual Scale
UWCH: University of Wisconsin Children’s Hospital Pain Scale
