# Supplementary material for: Pain assessment tools for use in infants: a meta-review
Source: BMC Pediatr. 2023 Jun 19;23:307. doi: 10.1186/s12887-023-04099-7 (PMC10278280; doi:10.1186/s12887-023-04099-7)
Supplement: Supplementary file 1 — Additionalfile1:SupplementaryTable1. Selection Criteria for the Systematic Review (PICOS). SupplementaryTable2. Databased searched, search dates and outputs . SupplementaryTable3. Database search strategies and outputs. SupplementaryTable4. List of search terms. SupplementaryTable5. List of Excluded reviews. SupplementaryTable6. List of included reviews. [file 12887_2023_4099_MOESM1_ESM.docx]

**Supplementary Tables**

**Supplementary Table 1: Selection Criteria for the Systematic Review (PICOS)**

|  | **Inclusion Criteria** | **Exclusion Criteria** |
| --- | --- | --- |
| **P** (participants) | Infant population | Studies on preterm, neonates and children < 12 months |
| **I** (intervention) | Pain experiences, including acute or chronic pain | - |
| **C** (comparison) | Pain assessment tools. | - |
| **O** (outcome) | The assessment of the proper pain scale, including details of validity, reliability, sensitivity and utility. | Studies which investigate pain on the basis of different scales. |
| **S** (study type) | Systematic literature review | Narrative review article, conference communication, case studies, editorials, diary studies.  Randomized trial study; Experimental study; Prospective study. |

**Supplementary Table 2: Databased searched, search dates and outputs**

| **Database** | **Number References** | **Search date** | **Results saved EndNote** |
| --- | --- | --- | --- |
| Medline | 15/1/2021 | 124 | Yes |
| Embase | 15/1/2021 | 46 | Yes |
| CINAHL | 15/1/2021 | 29 | Yes |
| Maternity & Infant Care (MIDIRS) | 15/1/2021 | 16 | Yes |
| EMCare | 15/1/2021 | 12 | Yes |
| Scopus | 15/1/2021 | 127 | Yes |
| Google Scholar | 15/1/2021 | 50 (first 50 results only) | Yes |

**Supplementary Table 3: Database search strategies and outputs**

**Medline**


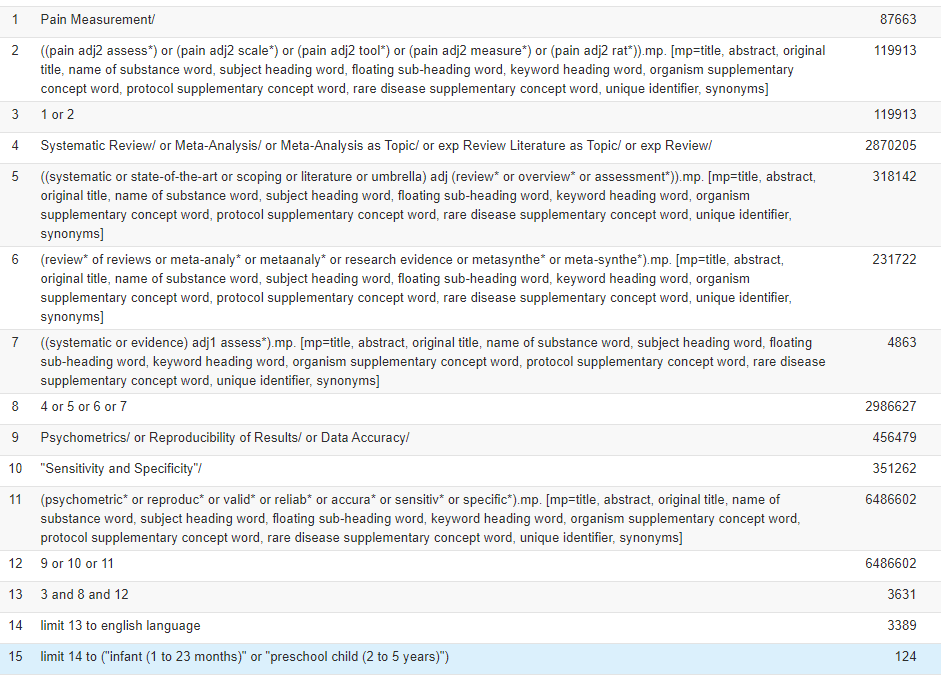


**Embase**


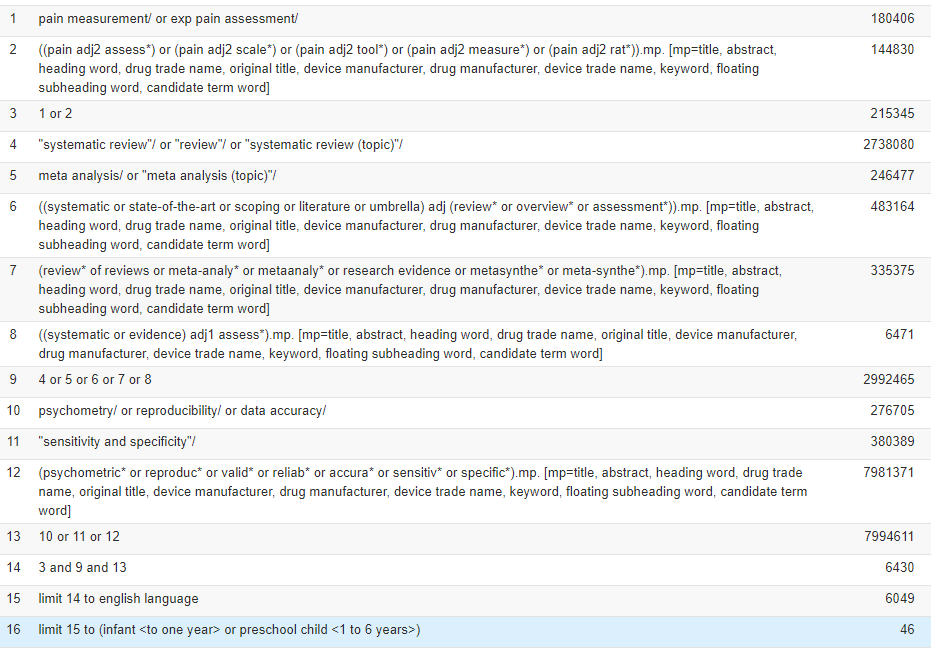


**CINAHL**


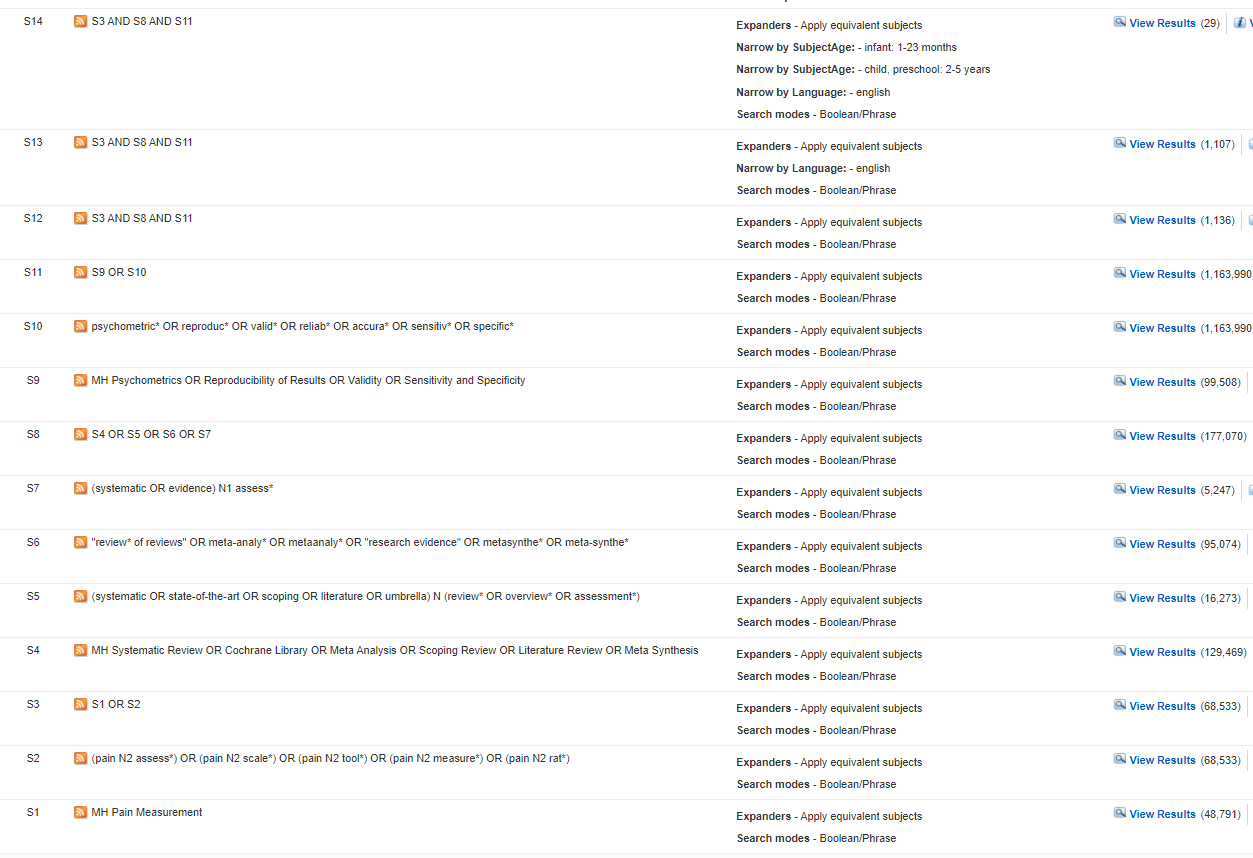


**Maternity & Infant Care (MIDIRS)**


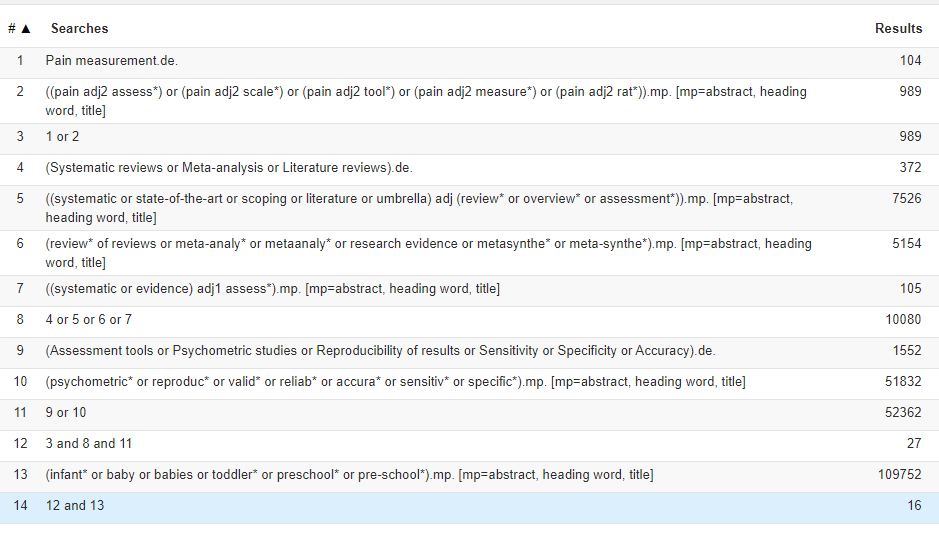


**EMCare**


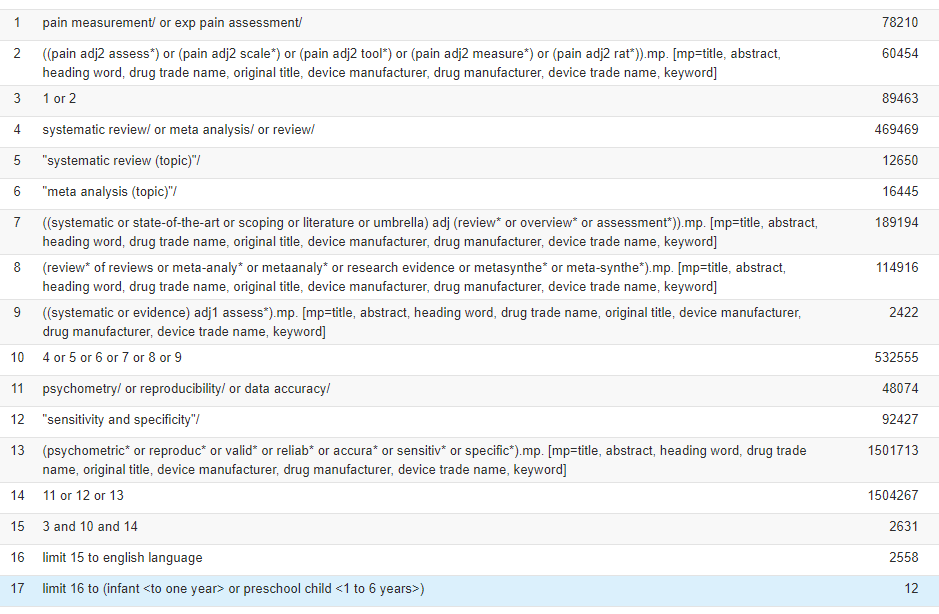


**Scopus**


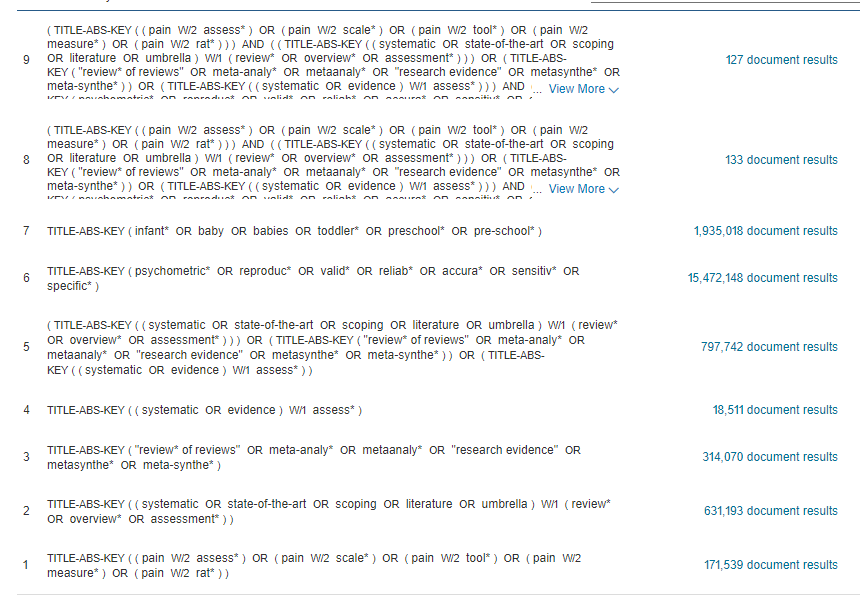


**Google Scholar**

(“pain (assessment|scale|tool|measure|rating”)(review|”meta analysis”|”meta synthesis”)(psychometrics|reproducibility|validity|reliability|accuracy|sensitivity|specificity)(infant|baby|toddler|preschool)


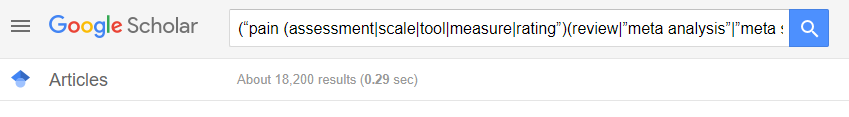


**Supplementary Table 4: List of search terms**

**OR**

|  | **Pain assessment** | **Systematic review** | **Tools** | **Infant/preschool** |
| --- | --- | --- | --- | --- |
| **Key Terms**  **Scopus** | (pain ADJ2 assess*) OR (pain ADJ2 scale*) OR (pain ADJ2 tool*) OR (pain ADJ2 measure*) OR (pain ADJ2 rat*) | (systematic OR state-of-the-art OR scoping OR literature OR umbrella) ADJ (review* OR overview* OR assessment*)  review* of reviews OR meta-analy* OR metaanaly* OR research evidence OR metasynthe* OR meta-synthe*  (systematic OR evidence) ADJ1 assess* | psychometric* OR reproduc* OR valid* OR reliab* OR accura* OR sensitiv* OR specific* | infant* OR baby OR babies OR toddler* OR preschool* OR pre-school*  LIMIT LANGUAGE – English |
| **MEDLINE** | Pain Measurement/ | Systematic Review/ OR Meta-Analysis/ OR Meta-Analysis as Topic/ OR  exp Review Literature as Topic/ OR exp Review/  [exp Review Literature as Topic = SR as Topic] | Psychometrics/ OR Reproducibility of Results/ OR Data Accuracy/  “Sensitivity and Specificity”/ | LIMIT AGE – Infant OR Preschool Child |
| **EMBASE** | pain measurement/ OR  exp pain assessment/ | systematic review/ OR “systematic review (topic)”/ OR meta analysis/ OR “meta analysis (topic)”/ OR review/ | psychometry/ OR reproducibility/ OR data accuracy/ OR “sensitivity and specificity”/ | LIMIT AGE – Infant OR Preschool Child |
| **CINAHL** | Pain Measurement | Systematic Review OR Cochrane Library OR Meta Analysis OR Scoping Review OR Literature Review OR Meta Synthesis | Psychometrics OR Reproducibility of Results OR Validity OR Sensitivity and Specificity | LIMIT AGE – Infant OR Child, Preschool |
| **MIDIRS** | Pain measurement | Systematic reviews OR Meta-analysis OR Literature reviews | Assessment tools OR Psychometric studies OR Reproducibility of results OR Sensitivity OR Specificity OR Accuracy | NO age filter – use keywords  NO English filter |
| **EMCARE** | pain measurement/ OR  exp pain assessment/ | systematic review/ OR meta analysis/ OR review/  systematic review (topic)/  meta analysis (topic)/ | psychometry/ OR reproducibility/ OR data accuracy/  sensitivity and specificity/ | LIMIT AGE – Infant OR Preschool Child |

**Supplementary Table 5. List of Excluded reviews.**

| **ID** | **Article** | **Reason for exclusion** |
| --- | --- | --- |
|  | McGrath, P. A. (1987). An assessment of children's pain: A review of behavioral, physiological, and direct scaling techniques. *Pain,* *31* (2), 147-176. | Narrative review. No data on psychometric properties of the tools. |
|  | Beyer, J. E., & Wells, N. (1989). The assessment of pain in children. *Pediatric Clinics of North America, 36*(4), 837-8554. | Narrative review. No data on psychometric properties of the tools. |
|  | McGrath, P. A. (1989). Evaluating a child's pain*. Journal of Pain and Symptom Management, 4* (4), 198-214. | Narrative review. No data on psychometric properties of the tools. |
|  | Hain, R. D. (1997). Pain scales in children: A review. *Palliative Medicine, 11* (5), 341-350. | Narrative review. No data on psychometric properties of the tools. |
|  | Gibbins, S., & Stevens, B. (2000). State of the art: Pain assessment and management in high-risk infants. *Pain Management Nursing, 1*(2),85-96. | Narrative review. No data on psychometric properties of the tools. |
|  | Zarbock, S. F. (2000). Pediatric pain assessment.  *Home care Provider, 5*(5), 181-184. | Full text couldn’t be obtained. |
|  | Summers, S. (2001). Evidence-based practice part 2: Reliability and validity of selected acute pain instruments. *Journal of Perianesthesia Nursing, 16*(1), 35-40. | Out of age limit assigned for the current study. |
|  | Stevens, B. J., & Franck, L. S. (2001). Assessment and management of pain in neonates. *Paediatric Drug,* *3*(7), 539-558. | Out of age limit assigned for the current study. |
|  | Van Dijk, M., Peters, J. W. B., Bouwmeester, N. J., & Tibboel, D. (2002). Are postoperative pain instruments useful for specific groups of vulnerable infants?. *Clinics in Perinatology, 29*(3):469-491. | Narrative review. Out of age limit assigned for the current study. |
|  | Solodiuk, Jean., & Curley, M. A. Q. (2003). Pain assessment in nonverbal children with severe cognitive impairments: the Individualized Numeric Rating Scale (INRS). *Journal of Pediatric Nursing, 18*(4),295-299. | Narrative review. No data on psychometric properties of the tools. |
|  | Ramelet, A., Abu-Saad, H. H., Rees, N., & McDonald, S. (2004). The challenges of pain measurement in critically ill young children: A comprehensive review. *Australian Critical Care, 17*(1):33-45. | Validity and reliability data reported but without further details useful for the current study purposes. |
|  | Ghai, B., Makkar, J., & Wig, Jyotsna. (2008). Postoperative pain assessment in preverbal children and children with cognitive impairment. *Paediatric Anaesthesia,18*(6),462-477. | Validity and reliability data reported but without further details useful for the current study purposes. |
|  | Correia, L. L., & Linhares, M. B. M. (2008). Assessment of the behavior of children in painful situations: Literature review*.  Journal de Pediatria, 84*(6),477-486. | Narrative review. No data on psychometric properties of the tools. |
|  | Stinson, J. N. (2009). Improving the assessment of pediatric chronic pain: Harnessing the potential of electronic diaries. *Pain Research & Management, 14*(1), 59-64. | No reported tools or psychometric data useful for the current study purposes. |
|  | Van Dijk, M., Valkenburg, A., Boerlage, A. A., Tibboel, D., & Veerkamp, J. S. (2009). Children with intellectual disabilities and pain perception: a review and suggestions for future assessment protocols. *European Archives of Paediatric Dentistry, 10*(2), 57-60. | Narrative review. No data on psychometric properties of the tools. |
|  | Valkenburg, A. J., Van Dijk, M., De Klein, A., Van den Anker, J. N., & Tibboel, D. (2010). Pain management in intellectually disabled children: Assessment, treatment, and translational research.  *Developmental Disabilities Research Reviews,* *16*(3), 248-257. | Validity and reliability data reported but without further details useful for the current study purposes. |
|  | Tomlinson, D., Von Baeyer, C. L., Stinson, J. N., & Sung, L. (2010). A systematic review of faces scales for the self-report of pain intensity in children. *Pediatrics,* *126*(5), e1168-e1198. | All reported tools were above age limit assigned for the current study. Validity and reliability data reported but without further details useful for the current study purposes. |
|  | Raeside, L. (2011). Physiological measures of assessing infant pain: A literature review. *British Journal of Nursing, 20*(21),1370-1376. | Narrative review of physiological measures. Below age limit assigned for the current study. No data on psychometric properties of the tools. |
|  | Van Dijk, M., & Tibboel, D. (2012). Update on pain assessment in sick neonates and infants. *Pediatric Clinics of North America, 59*(5), 1167-1181. | No data on psychometric properties of the tools. Below age limit assigned for the current study. |
|  | Jain, A. A., Yeluri, R., & Munshi, A. K. (2012). Measurement and assessment of pain in children: A review. *The Journal of Clinical Pediatric dentistry, 37*(2),125-136. | Narrative review. No data on psychometric properties of the tools. |
|  | Cong, X., McGrath, J. M., Cusson, R. M., & Zhang, D. (2013). Pain assessment and measurement in neonates: An updated review. *Advances in Neonatal Care, 13*(6), 379-395. | Below age limit assigned for the current study purposes. |
|  | Melo, G. M., Lélis, A.L., Moura, A. F., Cardoso, M. L., & Silva, V. M. (2014). Pain assessment scales in newborns: Integrative review. *Revista Paulista de Pediatria (English Edition),32*(4), 395-402. | Narrative review. No data on psychometric properties of the tools. |
|  | Hatfield, L. A., Ely, & Elizabeth, A. (2015). Measurement of acute pain in infants: A review of behavioral and physiological variables. *Biological Research for Nursing, 17*(1),100-111. | Narrative review of physiological and behavioural measures. Below age limit assigned for the current study. No data on psychometric properties of the tools. |
|  | Zamzmi, G., Goldgof, D., Kasturi, R., Sun, Y., & Ashmeade, T. (2016). Machine-based multimodal pain assessment tool for infants: A review. *arXiv preprint arXiv: 1607.00331, 0*(0). | Full text couldn’t be obtained. |
|  | Benoit, B., Martin-Misener, R., Newman, A., Latimer, M., & Campbell-Yeo, M. (2017). Neurophysiological assessment of acute pain in infants: A scoping review of research methods*. Acta Paediatrica (Oslo, Norway: 1992),* *106*(7),1053-1066. | No data on psychometric properties of the tools. Below age limit assigned for the current study. |
|  | Beltramini, A., Milojevic, K., & Pateron, D. (2017). Pain assessment in newborns, infants, and children. *Pediatric Annals, 46*(10), e387-e395. | Narrative review. No data on psychometric properties of the tools. |
|  | Zamzmi, G., Kasturi, R., Goldgof, D., Zhi, R., Ashmeade, T., & Sun, Y. (2017).  A review of automated pain assessment in infants: features, classification tasks, and databases. *IEEE Reviews in Biomedical Engineering,* *11*(0), 77-96. | Narrative review. No data on psychometric properties of the tools. Below age limit assigned for the current study purposes. |
|  | Andersen, R. D., Langius-Eklöf, A., Nakstad, B., Bernklev, T., & Jylli, L. (2017). The measurement properties of pediatric observational pain scales: A systematic review of reviews. International Journal of Nursing Studies, 73(0), 93-101. | Systematic review of systematic reviews |
|  | Hu, J., Modanloo, S., Squires, J. E., Harrold, J., & Harrison, D. (2019). The validity of skin conductance for assessing acute pain in infants: A scoping review. *The Clinical journal of pain,* *35*(8), 713-724. | Narrative review of skin conductance tools that are out of the scope of the current study purposes. |
|  | Cheng, D., Liu, D., Philpotts, L. L., Turner, D. P., Houle, T. T., Chen, L., Zhang, M., Yang, J., Zhang, W., & Deng, H. (2019). Current state of science in machine learning methods for automatic infant pain evaluation using facial expression information: study protocol of a systematic review and meta-analysis. *MJ Open, 9*(12), 1-8. | No tools were reported and no data on psychometric properties of the tools. |
|  | Meesters, N., Dilles, T., Simons, S., & Van Dijk, M. (2019). Do pain measurement instruments detect the effect of pain-reducing interventions in neonates? A systematic review on responsiveness. *Journal of Pain,* *20*(7),760-770. | No reported data on psychometric properties of the tools. |
|  | Loeffen, A. H., Stinson, J. N., Birnie, K. A., Van Dijk, M., Kulkarni, K., Rijsdijk, M., Font-Gonzalez, A., Dupuis, L. L., Van Dalen, E. C., Mulder, R. L., Campbell, F., Tissing, J. E., Van De Wetering, M. D., & Gibson, F. (2019). Measurement properties of instruments to assess pain in children and adolescents with cancer: A systematic review protocol. *Systematic Reviews, 8*(1), 2-8. | Narrative review restricted to children with cancer. No reported data on psychometric properties of the tools. |
|  | Miller-Hoover, S. R. (2019). Using valid and reliable tools for pain and sedation assessment in pediatric patients. *Critical Care Nurse, 39*(3), 59-66. | Reported tools and their psychometric data are very limited with no usefulness for the current study. |
|  | Greenfield, K., Holley, S., Schoth, D. E., Bayliss, J., Anderson, A. K., Jassal, S., Rajapakse, D., Fraser, L. K., Mott, C., Johnson, M., Wong, I., Howard, R., Harrop, E., & Liossi, C. (2020). A protocol for a systematic review and meta-analysis to identify measures of breakthrough pain and evaluate their psychometric properties. *BMJ Open*, *10*(3), 1-8. | No tools were reported and no data on psychometric properties of the tools. |
|  | Olsson, E., Ahl, H., Bengtsson, K., Vejayaram, D., Norman, E., Bruschettini, M., & Eriksson, M. (2020). The use and reporting of neonatal pain scales: a systematic review of randomized trials. *Pain,* *0*(0). | No tools were reported and no data on psychometric properties of the tools. |

**Supplementary Table 6: List of included reviews**

| **ID** | **Article** | **Notes on Inclusion** |
| --- | --- | --- |
|  | Duhn, L. J., & Medves, J. M. (2004). A systematic integrative review of infant pain assessment tools. *Advances in Neonatal care*, *4* (3), 126-140. | Meets inclusion criteria. Data on psychometric properties was reported. |
|  | Crellin, D., Sullivan, T. P., Babl, F. E., O'Sullivan, R., & Hutchinson, A. (2007). Analysis of the validation of existing behavioral pain and distress scales for use in the procedural setting. *Paediatric Anaesthesia, 17*(8), 720-733. | Meets inclusion criteria. Data on psychometric properties was reported. |
|  | Stapelkamp, C., Carter, B., Gordon, J., & Watts, C. (2011). Assessment of acute pain in children: Development of evidence-based guidelines. *International Journal of Evidence-Based Healthcare, 9*(1), 39-50. | Narrative review. No data on psychometric properties of the tools. |
|  | Crosta, Q. R., Ward, T. M., Walker, A. J., & Peters, L. M. (2014). A review of pain measures for hospitalized children with cognitive impairment. *Journal for Specialists in Pediatric Nursing: JSPN, 19*(2), 109-118. | Meets inclusion criteria. Data on psychometric properties was reported. |
|  | Kingsnorth, S., Orava, T., Provvidenza, C., Adler, E., Ami, N., Gresley-Jones, T., Mankad, D., Slonim, N., Fay, L., Joachimides, N., Hoffman, A., Hung, R., & Fehlings, D. (2015). Chronic pain assessment tools for cerebral palsy: A systematic review. *Pediatrics , 136*(4), e947-e960. | Meets inclusion criteria. Data on psychometric properties was reported. |
|  | Crellin, D. J., Harrison, D., Santamaria, N., & Babl, F. E. (2015). Systematic review of the Face, Legs, Activity, Cry and Consolability scale for assessing pain in infants and children: Is it reliable, valid, and feasible for use?. *Pain*, *156*(11), 2132-2151. | Meets inclusion criteria. Data on psychometric properties was reported. |
|  | Bai, J., & Jiang, N. (2015). Where Are We: A Systematic evaluation of the psychometric properties of Pain Assessment Scales for use in Chinese children. *Pain Management Nursing, 16*(4), 617-631. | Meets inclusion criteria. Data on psychometric properties was reported. |
|  | Maaskant, J., Raymakers-Janssen, P., Veldhoen, E., Ista, E., Lucas, C. & Vermeulen, H. (2016). The clinimetric properties of the COMFORT scale: A systematic review. *European Journal of Pain, 20*(10), 1587-1611. | Meets inclusion criteria. Data on psychometric properties was reported. |
|  | Crellin, D. J., Babl, F. E., Santamaria, N., & Harrison, D. (2018). A systematic review of the psychometric properties of the Modified Behavioral Pain Scale (MBPS). *Journal of Pediatric Nursing, 40*(0), 14-26. | Meets inclusion criteria. Data on psychometric properties was reported. |
|  | Giordano, V., Edobor, J, Deindl, P., Wildner, B., Goeral, K., Steinbauer, P., Werther, T., Berger, A., & Olischar, M. (2019). Pain and sedation scales for seonatal and pediatric patients in a preverbal stage of development: A systematic review. *JAMA* *Pediatr, 173*(12), 1186-1197. | Meets inclusion criteria. Data on psychometric properties was reported. |
